# Effects of different mesh materials on complications after prophylactic placement for stoma formation: a systematic review and network meta-analysis

**DOI:** 10.1007/s10029-024-03068-y

**Published:** 2024-06-15

**Authors:** C. Meng, Q. Wei, L. Sun, X. Zhang, Y. Liu, J. Gao, P. Wei, Z. Yang, H. Yao, Z. Zhang

**Affiliations:** Department of General Surgery, Beijing Friendship Hospital, Capital Medical University, State Key Lab of Digestive Health, National Clinical Research Center for Digestive Diseases, Beijing, China

**Keywords:** Parastomal hernia, Mesh material, Network meta-analysis, Prophylactic placement, Mesh

## Abstract

**Purpose:**

We primary aimed to synthesise the available data, assess the effectiveness of different mesh materials in prophylactic mesh placement, and rank these materials according to the incidence of parastomal hernia (PSH) and other stoma complications.

**Method:**

This network meta-analysis performed a systematic review and meta-analysis according to the Preferred Reporting Items for Systematic Review and Meta-Analysis statement. Four databases were searched for randomised controlled trials of prophylactic mesh placement. The aggregated results were performed in the STATA routine for Bayesian hierarchical random effects models.

**Result:**

Thirteen randomised controlled trials from 1203 articles, met the inclusion criteria, including 681 cases without meshes, 65 cases with mesh material of xenogeneic acellular dermis (porcine/bovine), 27 cases with polypropylene/PG910, 114 cases with polypropylene/polyglecaprone (Monocryl), 117 cases with polypropylene/cellulose (ORC), 233 cases with polypropylene, and 35 cases with polypropylene/PVDF. In network A, compared with no mesh, only polypropylene (RR 0.24, 95% CI 0.04–0.80) were significantly associated with a reduction in the incidence of PSH. In network B, no statistical difference regarding stoma complications was found between mesh and no mesh.

**Conclusion:**

Based on the network meta-analysis and ranking results, the polypropylene mesh material exhibited the best performance. However, this conclusion needs to be confirmed with larger sample sizes and high-quality randomised controlled trials.

**Supplementary Information:**

The online version contains supplementary material available at 10.1007/s10029-024-03068-y.

## Introduction

Parastomal hernia (PSH) is a common complication of abdominal stomas. According to the guidelines of the European Hernia Society, the incidence of PSH is > 30% at 12 months after stoma and > 40% at two years after surgery. After prolonged follow-up, the incidence rate is > 50% [1, 2]. The highest incidence rate is for end colostomy (4–48%), followed by loop colostomy (0–36%), and end ileostomy (2–28%) [3]. Parastomal hernias affect the quality of life of patients. The common clinical symptoms include pain and leakage. In addition, 15% of patients with PSH experience complications such as obstruction, perforation, and strangulation [4]. Studies have shown that 20–30% of PSH require surgical treatment, whereas the postoperative recurrence rate is > 30% [5].

The high prevalence of PSH and high rate of recurrence after surgical repair highlight the importance of primary prevention. Recently, several surgical procedures have been proposed to prevent PSH formation after primary surgery, including transrectus versus pararectus, transperitoneal versus extraperitoneal, and correct sizing of the trephine [6]. However, none of these treatments reduced the incidence of PSH. Therefore, attention has been given to the use of prophylactic meshes to prevent PSH. Over the past 20 years, eight small and two large randomised controlled trials (RCTs) have been published [7–12]. Although the location of the mesh, choice of the mesh material, and surgical approach varied, the data from these studies showed encouraging results.

With the development of material technology, synthetic, composite, and biological meshes have been developed. Synthetic meshes mainly include polypropylene and polyvinylidene fluoride (PVDF), which are non-absorbable and have high tensile strength, which can significantly reduce the occurrence of parastomal hernias, however, polypropylene meshes may cause severe adhesions, erosions, or incision infections, and other complications [13, 14]. A composite mesh is a special synthetic mesh based on polypropylene and includes polypropylene/polyglecaprone, polypropylene/PG910, and polypropylene/cellulose. This reduces the stickiness of the abdominal contents to the mesh; however, this effect decreases over time [1]. The biological mesh is made of bovine or porcine decellularised collagen matrix that can be slowly degraded and replaced by fibrous tissue; its greatest advantage is that it can be used in polluted environments [15].

Currently, the published RCTs are controlled studies between mesh and no-mesh groups, and controlled studies between different types of meshes are scarce. Recently, systematic reviews discussed the efficacy of synthetic and biological mesh analyses. In a meta-analysis, Patel et al. included the results of nine groups (two groups of biological meshes) and showed that the placement of biological and synthetic meshes at the time of stoma can reduce the occurrence of parastomal hernia; however, the synthetic mesh was more effective (*P* = 0.01) [16]. Another study showed that the type of mesh prophylactically placed at the time of stoma placement did not affect the incidence of parastomal hernias [17]. Therefore, the primary objective of this study was to perform a systematic review and meta-analysis of all eligible RCTs to assess the effects of different mesh types on the incidence of PSH and stoma complications.

## Methods

### Literature search and search strategy

This systematic review and network meta-analysis was performed according to the Preferred Reporting Items for Systematic Review and Meta-Analysis (PRISMA) [18] and Assessing the Methodological Quality of Systematic Reviews (AMSTAR) guidelines [19]. The protocol was registered in PROSPERO (CRD42023432191).

The following databases were searched from database inception to November 2022: EMBASE, Medline, Cochrane, and Cochrane Central Register of Controlled Trials (CENTRAL). We manually retrieved unpublished grey literature and searched for references in the original text to avoid omissions as much as possible. The search strategy was conducted using the following terms: ‘mesh’, ‘prevention’, ‘parastomal hernia’, and ‘randomised controlled trial’. The full search terms and an example of the search strategy are provided in Supplementary File S1.

### Eligibility criteria

RCTs comparing the prophylactic placement of a mesh (synthetic, composite, or biological) with no mesh for ostomy (colostomy or ileostomy) were eligible for inclusion. We did not consider the sample size, publication date, surgical technique, or language of the publication. Relevant observational and single-arm studies were also excluded. Case reports, reviews, meta-analyses, letters to the editor, and editorials were excluded. Finally, RCTs with follow-up times > 12 months were prioritised.

### Data extraction and management

Data were independently extracted by two investigators using predesigned standardised forms. The extracted data included study characteristics (authors, year of publication, and study design), patient demographics (age, sex, and body mass index), treatment characteristics (surgical method, mesh type, and mesh location), PSH diagnostic method, number of PSH, number of stoma-specific complications, and follow-up time. Most of the included studies had mesh placement in "Sublay". In the sublay group, the mesh was placed in the position, dorsal to the rectus abdominis muscle and anterior to the posterior rectus sheath. This position can avoid direct contact between the mesh and intestinal loops, thereby avoiding adhesions; at the same time, the mesh is placed behind the rectus abdominis muscle, which can evenly distribute intra-abdominal pressure to the entire abdominal wall, which conforms to mechanical principles and can reduce the possibility of hernia. In addition, the mesh adheres closely to the muscles, which facilitates the growth and attachment of granulation tissue, accelerates the "abdominal wall" process of the mesh, and increases the strength of the abdominal wall.

The primary outcome was the incidence of PSH (clinical and/or radiographic) at the last follow-up, which was analysed separately according to surgical approach and mesh location. The secondary outcomes included the incidence of other stoma-related complications mainly include skin problems/stoma necrosis/stoma prolapse/stoma stenosis/stoma bleeding and stoma infection.

### Risk of bias and quality assessment

Two authors (CM and QW) independently assessed the risk of bias in the included studies using the Cochrane Risk of Bias Tool for Randomised Controlled Trials 2.0 [20], with a ‘low’, ‘unclear’, or ‘high’ risk of bias grading level. Disagreements between the two researchers were resolved by consensus after discussions with a third researcher (LS).

### Statistical analysis

R4.1 software was used to call the ‘netmeta’ package to construct a Markov chain Monte Carlo (MCMC) for the Bayesian network meta-analysis. Outcomes assessed in this network meta-analysis involving both networks included the occurrence of PSH and other ostomy complications. Among all references, 13 studies reported the occurrence of PSH, and 11 studies reported the occurrence of other ostomy complications (Supplementary File S2). We named the networks assessing PSH and other stoma complications A and B, respectively, which involved six treatment modalities and were directly compared in 13 and 11 randomized controlled trials, respectively. To explore the possible sources of heterogeneity in the impact of different meshes on the occurrence of PSH, a subgroup analysis was performed on two possible confounding factors: surgical procedure and mesh location. Considering that the number of included studies is large, the time span is large, and the heterogeneity between studies may be large, we therefore choose a random effects model with wider applicability. In this study, a random-effects model was adopted, and four Markov chains were set 10,000 times for annealing and iterated 50,000 times to achieve model convergence. The relative risk (RR) and odds ratio (OR) was selected as the dichotomous effect size both with a 95% confidence interval (CI). As the included RCTs were conducted in different settings and countries, we decided to report the results using a random-effects model. In this network meta-analysis, we did not have closed loops in the network; thus, we could not assess the consistency assumption. The surface under the cumulative ranking (SUCRA) was used to rank the pros and cons of the interventions, and the value range was 0–1; the larger the value, the better the treatment effect [21]. Stata 14.0 software (StataCorp, College Station, TX) was used to draw network evidence graphs and correct funnel plots [22]. The inspection level was set to α = 0.05.

## Results

### Study selection and characteristics

**Fig. 1:**
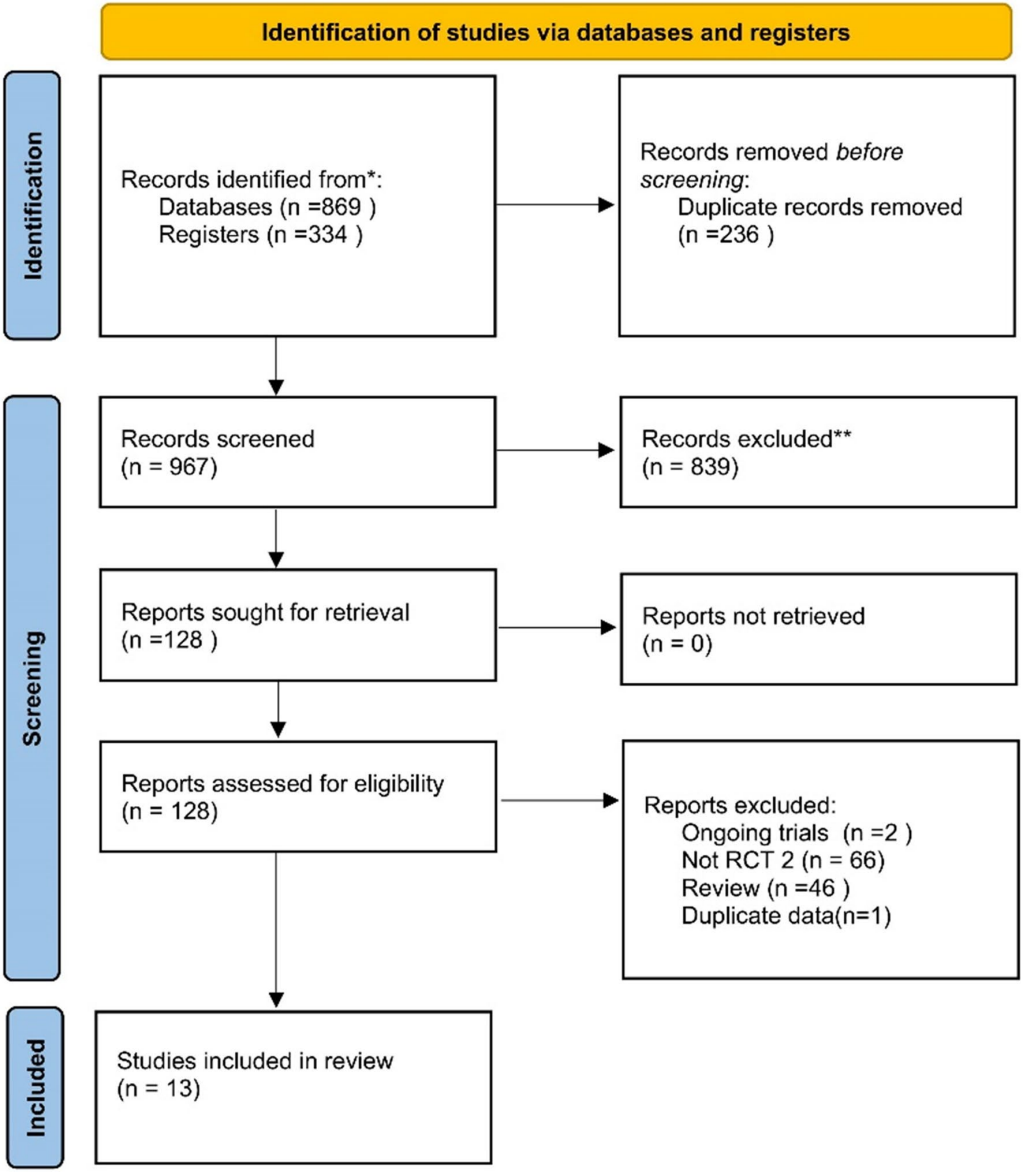
Literature search flowchart. *Consider, if feasible to do so, reporting the number of records identified from each database or register searched (rather than the total number across all databases/registers). **If automation tools were used, indicate how many records were excluded by a human and how many were excluded by automation tools. From: Page MJ, McKenzie JE, Bossuyt PM, Boutron I, Hoffmann TC, Mulrow CD, et al. The PRISMA 2020 statement: an updated guideline for reporting systematic reviews. BMJ 2021;372:n71. https://doi.org/10.1136/bmj.n71. For more information, visit: http://www.prisma-statement.org/

The preliminary search resulted in 1203 articles. Of these, 236 were excluded due to duplication. Two independent researchers screened the titles and abstracts, and 839 articles were excluded because they did not match the topic. A total of 128 articles were included in the next step. The agreement rate between the two researchers was 95%. Ultimately, 13 RCTs met the eligibility criteria [7, 9–12, 23–30]. For this network meta-analysis, the literature search flowchart is shown in Fig. 1. These RCTs reported the occurrence of parastomal hernia during the follow-up period of patients with prophylactic mesh or no mesh, and the follow-up time was ≥ 12 months. The characteristics of the included RCTs are shown in Table 1.

**Table 1:** Characteristics of included RCTs

Author (year)	Centre (country)	Randomised sample size (mesh/no mesh groups)	Arm	Gender ratio (M:F)	Mean age (± SD), years	Mean BMI (± SD), kg/m^2^	Diseases	Type of ostomy	Mesh type	Approach	Mesh position	PSH detection	PSH (n)	Incidence of stome related complications(n)	follow-up (months)
Ammond et al. [12] (2008)	Single centre (UK)	20(10/10)	Mesh No mesh	4: 6 3: 7	42.6(N/A) 50.0(N/A)	26.3(N/A) 26.3(N/A)	Cancer (15%)/Benign (85%)	NR	Xenogenic acellular dermis (porcine/bovine)	Open	Preperitoneal	Clinical, US	0 3	3 5	12
Janes et al. [7] (2009)	Single centre (Sweden)	54(27/27)	Mesh No mesh	15: 12 16: 11	70.0(N/A) 70.0(N/A)	26.0(N/A) 27.0(N/A)	Cancer (87%)/Benign (13%)	End colostomy only	Polypropylene/PG910	Open	Sublay	Clinical	1 13	NR	65.2
Serra-Aracil et al. [9] (2009)	Single centre (Spain)	54(27/27)	Mesh No mesh	19: 5 16: 8	67.5(± 8.8) 67.2(± 9.7)	25.6(± 2.9) 27.3(± 3.5)	Cancer	End colostomy only	Polypropylene/polyglecaprone (Monocryl)	Open	Sublay	Clinical, CT	6 12	3 1	29
Lopez-Cano et al. [10] (2012)	Single centre (Spain)	36(19/17)	Mesh No mesh	11: 8 7: 10	72.2(± 7.6) 65.9(± 13.9)	26.3(± 3.2) 27.5(± 4.7)	Cancer	End colostomy only	Polypropylene/cellulose (ORC)	Lap	Intraperitoneal	CT	9 15	2 2	12
Fleshman et al. [23] (2014)	Multicentre (USA)	113(55/58)	Mesh No mesh	30: 25 29: 29	60.3(± 13.6) 59.1(± 14.4)	26.2(± 4.6) 24.7(± 4.1)	Cancer (47%)/Benign (53%)	Ileostomy(37%) Colostomy(63%)	Xenogenic acellular dermis (porcine/bovine)	Both	Sublay	Clinical, CT	6 7	6 8	24
Tarcoveanu et al. [11] (2014)	Single centre (Romania)	42(20/22)	Mesh No mesh	NR	NR	NR	Cancer	End colostomy only	Polypropylene	Open	Sublay	Clinical, US	0 6	NR	20
Lambrecht et al. [24] (2015)	Multicentre (Norway)	58(32/26)	Mesh No mesh	22: 10 21: 5	64.0(± 4.0) 63.0(± 4.1)	24.6(± 0.6) 25.5(± 0.8)	Cancer	End colostomy only	Polypropylene	Open	Sublay	Clinical, CT	2 12	3 5	36/48
Lopez-Cano et al. [25] (2016)	Multicentre (Spain)	52(24/28)	Mesh No mesh	21: 3 16: 8	70.5(± 9.5) 67.3(± 13.6)	25.3(± 2.8) 26.9(± 4.4)	Cancer	End colostomy only	Polypropylene/polyglecaprone (Monocryl)	Lap	Intraperitoneal	CT	6 18	10 6	26
Brandsma et al. [8] (2017)	Multicentre (Netherlands)	133(67/66)	Mesh No mesh	38: 29 37: 29	63.5(N/A) 63.0(N/A)	26.8(N/A) 26.5(N/A)	Cancer (88%)/Benign (12%)	End colostomy only	Polypropylene	Open	Sublay	Clinical, CT	3 16	15 23	12
Odensten et al. [27] (2019)	Multicentre (Sweden)	232(114/118)	Mesh No mesh	74: 40 62: 56	69.7(N/A) 69.9(N/A)	26.1(N/A) 26.3(N/A)	Cancer (91%)/Benign (9%)	End colostomy only	Polypropylene	Open	Sublay	Clinical, CT	33 36	7 9	12
Makarainen-Uhlback et al. [28] (2020)	Multicentre (Finland)	70(35/35)	Mesh No mesh	18: 17 19: 16	67.1(± 10.7) 65.1(± 11.7)	26.2(± 4.6) 25.4(± 4.3)	Cancer	End colostomy only	Polypropylene/PVDF	Lap	keyhole	Clinical, CT	9 16	5 7	65
Prudhomme et al. [29] (2021)	Multicentre (France)	199(98/101)	Mesh No mesh	57: 41 57: 44	67.2(± 12.4) 70.5(± 11.1)	25.6(± 4.6) 24.8(± 4.7)	Cancer (86%)/Benign (14%)	End colostomy only	Polypropylene/cellulose (ORC)	Both	Sublay	Clinical, CT	30 28	37 32	24
Correa Marinez et al. [30] (2021)	Multicentre (Sweden/Denmark)	209(63/146)	Mesh no mesh	42: 21 80: 66	65.0(N/A) 63.6(N/A)	26.3(N/A) 28.2(N/A)	Cancer (84%)/Benign (16%)	End colostomy only	Polypropylene/polyglecaprone (Monocryl)	Both	Sublay	Clinical, CT	23 56	11 35	12

(*NR* No report, *CT* computed tomography, *US* ultrasound)

### Risk of bias in studies

**Fig. 2:**
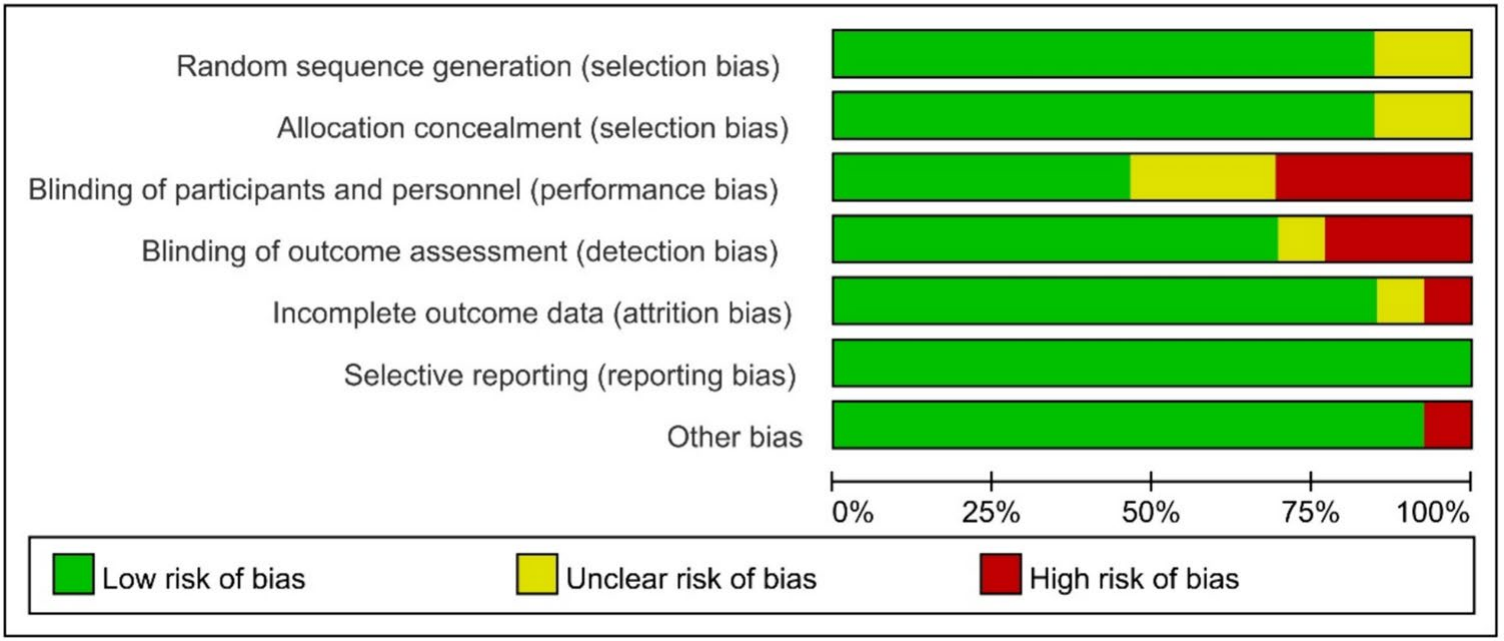
Methodological quality assessment

The quality of the research included in this study was inherently high. In addition, all RCTs were considered to have a low risk of selection and reporting biases (n = 13). The risk of overestimating effectiveness due to selective publication is low. The risk of performance bias was unclear in most RCTs (n = 6) because of the impossibility of blinding participants and staff. The included studies all provided the materials and manufacturers of the meshes used, and there was no limitation the data completeness of this study. The data we extracted were mostly basic, so there was little chance of underreporting in the original studies hampering the evaluation and the risk of attrition, reporting, and unknown deviations was low. Finally, 11 of the 13 RCTs were judged to be at a low risk of bias, while two RCTs were judged to be at a high risk of bias. The methodological assessment was subject to an overall low risk of bias and was of very good quality. The methodological quality assessment is shown in Fig. 2.

### Network meta-analysis

**Fig. 3:**
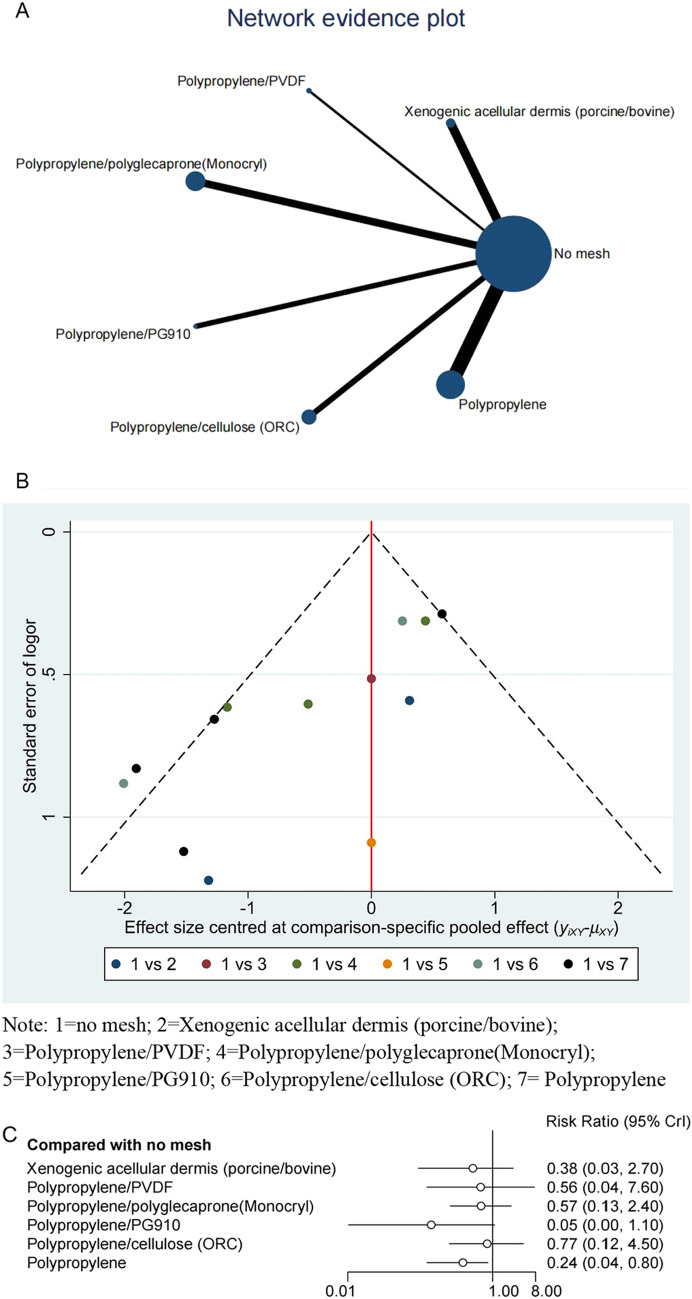
Network plots for networks A. Note: 1 = No mesh; 2 = Xenogenic acellular dermis (porcine/bovine); 3 = Polypropylene/PVDF; 4 = Polypropylene/polyglecaprone(Monocryl); 5 = Polypropylene/PG910; 6 = Polypropylene/cellulose (ORC); 7 = Polypropylene

Network A included 13 RCTs that compared the following six mesh materials: xenogeneic acellular dermis (porcine/bovine), polypropylene/PVDF, Polypropylene/ polyglecaprone (Monocryl), polypropylene/PG910, polypropylene/cellulose (ORC), and polypropylene (Fig. 3A). In this network, compared with no mesh, only polypropylene (RR = 0.24, 95% CI 0.04–0.80) were significantly associated with a reduction in the incidence of PSH. Among them, polypropylene is associated with lower rates of parastomal hernia compared to other mesh materials. In terms of ranking, polypropylene/PG910 and polypropylene were the best mesh materials in this network. The p-values for these materials were 90.9% and 67.1%, respectively (Table 2). The publication bias funnel plot was drawn according to the incidence of PSH, and the bottom panel was asymmetrical, indicating publication bias in this study (Fig. 3B). After the sensitivity analysis eliminated individual studies, it was observed that the combined effect size remained statistically significant, and the direction of the forest plot results did not change (Fig. 3C).

**Table 2:** Ranking of the merits and demerits of different mesh materials under different outcome indexes

intervention method	SUCRA(%)	
	PSH	Stoma related complications
Xenogenic acellular dermis (porcine/bovine)	43.1	68.6
Polypropylene/PVDF	45.8	63.3
Polypropylene/polyglecaprone (Monocryl)	45.7	26.2
Polypropylene/PG910	90.9	NA
Polypropylene/cellulose (ORC)	43.3	26.6
Polypropylene	67.1	76.2

(*PSH* parastomal hernia, *SUCRA* surface under the cumulative ranking)

**Fig. 4:**
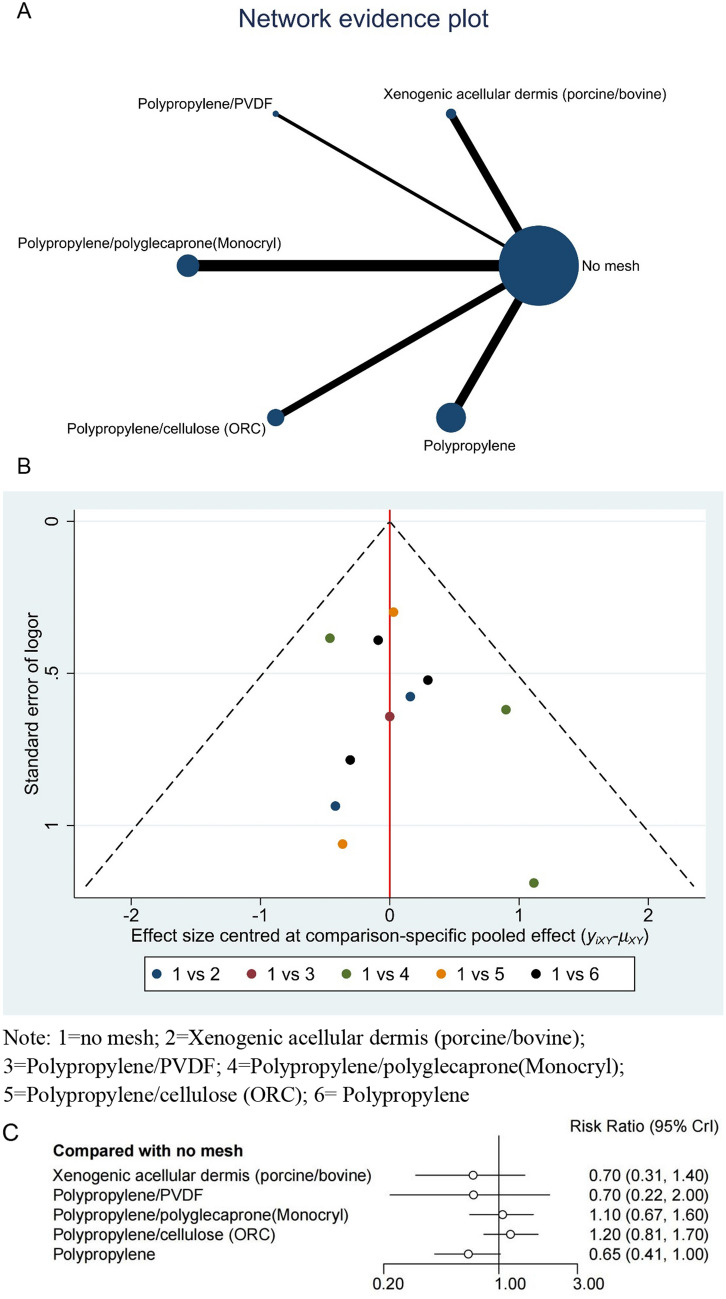
Network plots for networks B. Note: 1 = no mesh; 2 = Xenogenic acellular dermis (porcine/bovine); 3 = Polypropylene/PVDF; 4 = Polypropylene/polyglecaprone(Monocryl); 5 = Polypropylene/cellulose (ORC); 6 = Polypropylene

Network B included 11 RCTs on the following five treatments: xenogeneic acellular dermis (porcine/bovine), polypropylene/PVDF, polypropylene/Polyglecapro-ne (Monocryl), polypropylene/cellulose (ORC), and polypropylene (Fig. 4A). The network reports the results using a random-effects model. In this network, none of the meshes showed a significant advantage in reducing complications compared to no mesh. The analysis results indicate only polypropylene may have a potential advantage in preventing stoma-related complications compared with the no-mesh group (RR = 0.65, 95% CI 0.041–1.00). The comparison results for all meshes and no mesh in network B are listed in Table 2. The results showed that polypropylene and xenogeneic acellular dermis (porcine/bovine) mesh materials ranked first and second, respectively, in preventing stoma complications, and the effects of publication bias and small study sizes were assessed using corrected funnel plots (Fig. 4B). The funnel plot of the network was roughly symmetrical, suggesting no publication bias owing to small study effects. The forest plot of the meta-analysis results of Network B is shown in Fig. 4C.

### Subgroup analyses

**Fig. 5:**
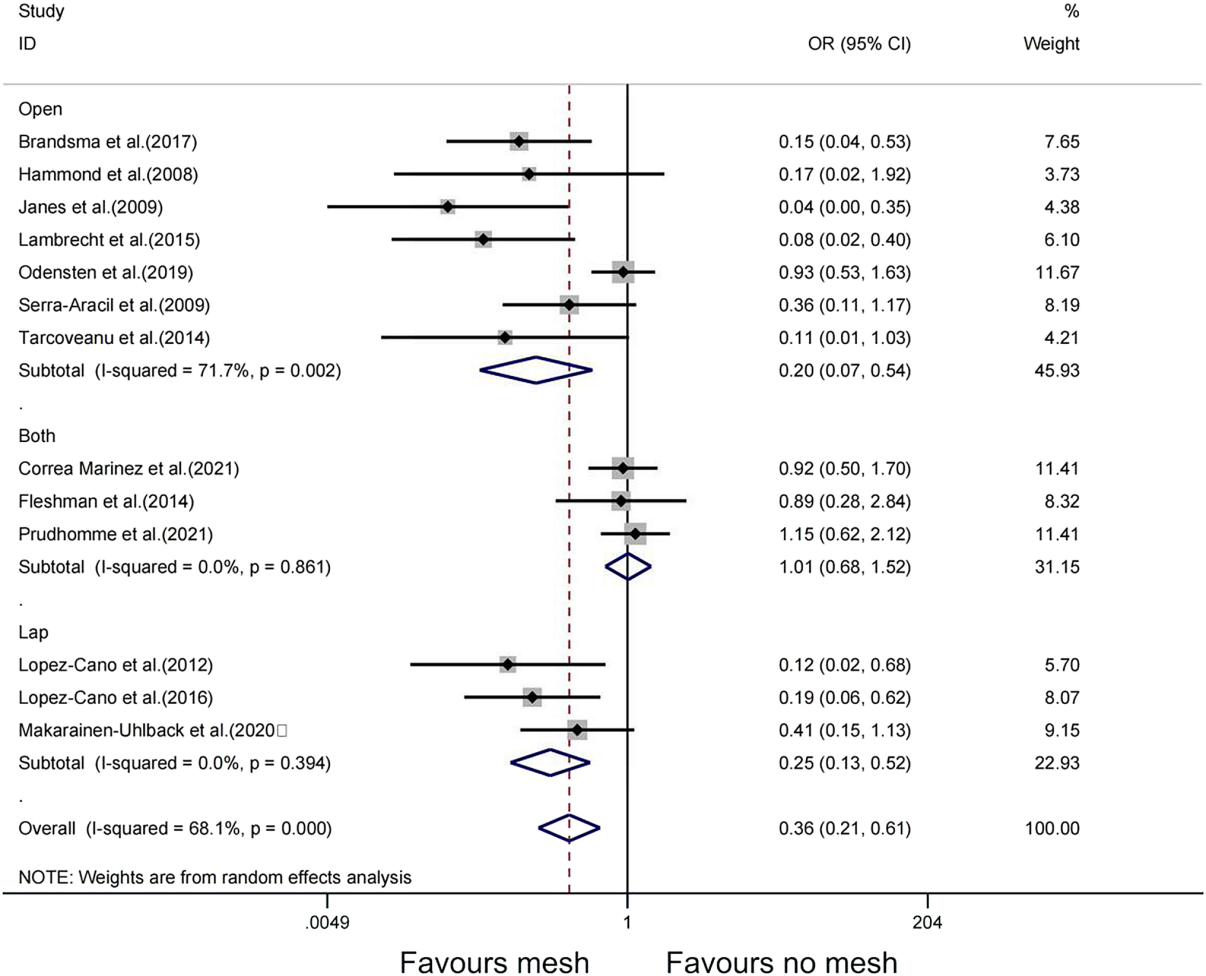
Subgroup analysis of the effect of surgical methods on PSH

**Fig. 6:**
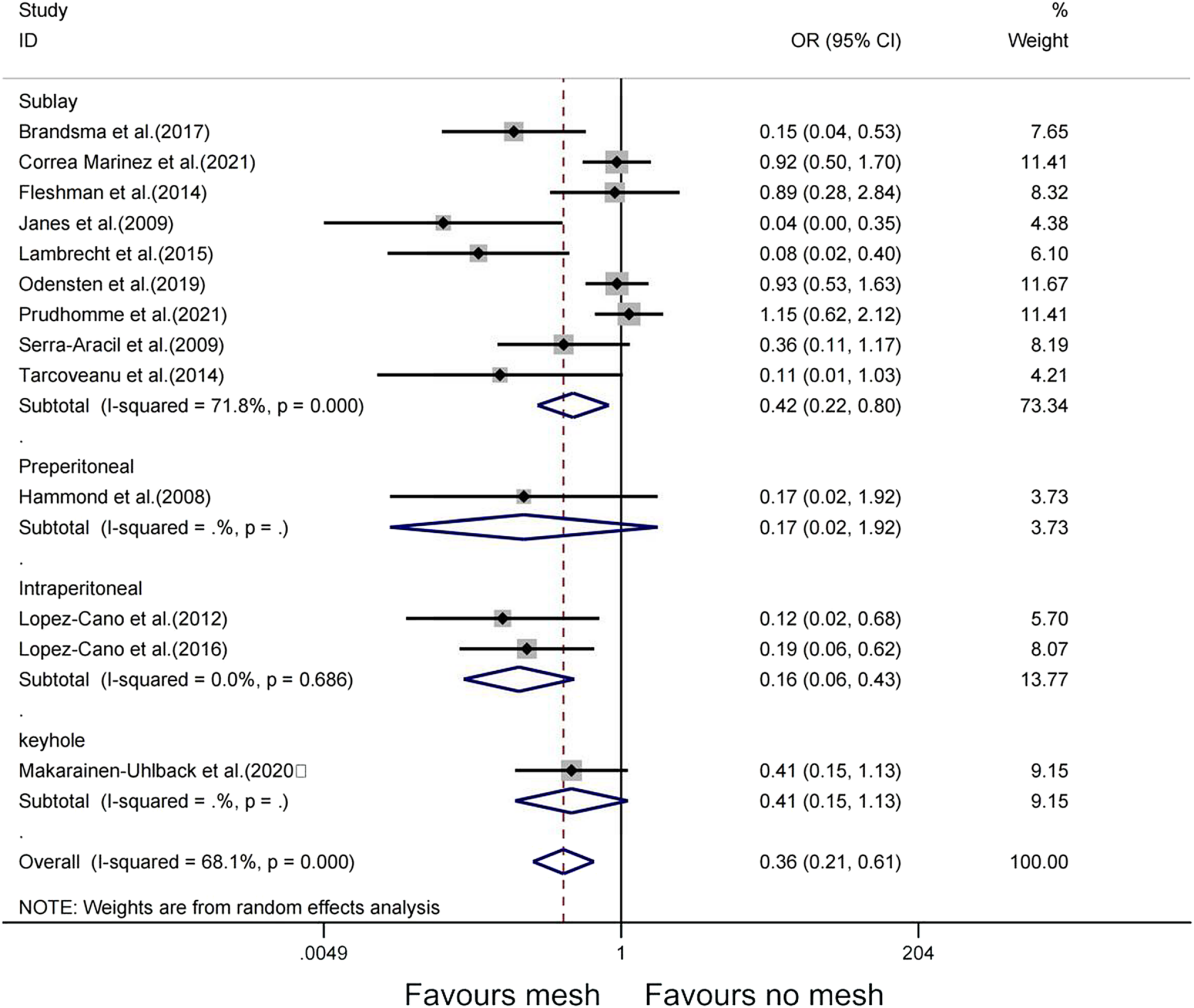
Subgroup analysis of the effect of stoma placement on PSH

Due to the statistical heterogeneity of the included studies, a subgroup analysis was used to explore the possible sources of heterogeneity of different meshes affecting the occurrence of PSH. Subgroup analyses were performed for two possible confounding factors: surgical procedure and mesh location. The laparoscopic subgroup (OR = 0.25, 95% CI 0.13–0.52, *P* = 0.394), open subgroup (OR = 0.20, 95% CI 0.07–0.54, *P* = 0.002), both subgroups (OR = 1.01, 95% CI 0.68–1.52,* P* = 0.861), 95% CI overlap between subgroups, and interaction analysis showed that a significant interaction between the combined effect size of the subgroup and the factors of different surgical methods (*P* < 0.05), and different surgical methods may be an important contributing factor to the source of heterogeneity (Fig. 5). Combined with the above results, both the laparoscopic subgroup and the open subgroup were associated with lower PSH rates compared with the no-mesh group. Comparison between the two groups showed that the open subgroup was associated with lower PSH rates than the laparoscopic subgroup (OR = 0.20, 95% CI 0.07–0.54; OR = 0.25, 95% CI 0.13–0.52). The difference is more significant (P = 0.002). However, high heterogeneity was observed among the studies (I^2^ = 0.68, P = 0.000). For mesh position, the sublay subgroup (OR = 0.42, 95% CI 0.22–0.80, *P* < 0.05), intraperitoneal subgroup (OR = 0.16, 95% CI 0.06–0.43,* P* = 0.686), 95% CI overlap, and interaction analysis between groups showed that a significant interaction between the combined effect size of subgroups and the location of different meshes (*P* < 0.05). In this analysis, we found that both the sublay subgroup and the intraperitoneal subgroup were associated with lower PSH rates compared with the no-mesh group (OR = 0.42, 95% CI 0.22–0.80; OR = 0.16, 95% CI 0.06–0.43). However, the difference between the sublay subgroup and the no mesh group was more significant (P < 0.001). Similarly, different meshes may also be important factors contributing to heterogeneity (Fig. 6). In addition, to further analyze the sources of heterogeneity, this study divided the mesh into groups according to the inside and outside of the peritoneum: extraperitoneal group (preperitoneal, sublay); intraperitoneal group (keyhole, intraperitoneal). Interaction analysis of the extraperitoneal subgroup (RR = 1.02, 95% CI 0.98–1.08, P = 0.725, I^2^ = 0%) and intraperitoneal subgroup (RR = 0.97, 95% CI 0.86–1.11, P = 0.431, I^2^ = 0%) showed that there was no significant interaction between the combined effect size of the subgroup and the factors of different methods of mesh placement (Supplementary Figure S1).

Due to the inconsistent follow-up time of the included studies, we divided the studies into studies with follow-up time ≤ 24 months and > 24 months for subgroup analysis. However, we did not find heterogeneity due to different follow-up times. The follow-up time ≤ 24 months subgroup (RR = 1.03, 95% CI 0.97–1.08, P = 0.887, I^2^ = 0%), follow-up time > 24 months subgroup (RR = 0.99, 95% CI 0.89–1.09, P = 0.352, I^2^ = 8.1%). interaction analysis showed that there was no significant interaction between the combined effect size of the subgroup and the factors of different follow-up time (P = 0.807) (Supplementary Figure S2).

### Sensitivity analysis

Results of sensitivity (studies with sample size of each arm greater than or equal to 50 patients) are summarized in Supplementary Figure S3 and S4. The sensitivity analysis of Network A finally included only two mesh materials: Xenogenic acellular dermis (porcine/bovine) and Polypropylene/polyglecaprone (Monocryl). The results showed that there was no significant difference compared with no mesh (RR = 0.77, 95% CI 0.27–2.1; RR = 0.71, 95% CI 0.36–1.3), which is consistent with our previous analysis results. The sensitivity analysis of Network B finally included only two mesh materials: Polypropylene/cellulose (ORC) and Polypropylene. The results showed that there was no significant difference compared with no mesh in terms of complications (RR = 1.20, 95% CI 0.82–1.80; RR = 0.68, 95% CI 0.41–1.1), which is consistent with our previous analysis results.

## Discussion

In this network meta-analysis, we compared the effects of different mesh materials on prophylactic mesh placement, including the incidence of PSH and other stoma complications. Furthermore, the mesh materials in the network were ranked using SUCRA. In our study, we observed that all mesh materials tended to reduce the incidence of PSH. However, owing to the small sample size, only polypropylene material had a statistically significant preventive effect on reducing the incidence of PSH, and no significant difference existed between the other materials and the control group. Studies have shown that polypropylene mesh is currently the most widely used mesh [31], and its large size is conducive to the growth of collagen fibres into the mesh, which can be fused with fibrous tissue. It can significantly reduce the occurrence of PSH owing to its non-absorbability and large pore size characteristics. In addition, in the analysis of other stoma-related complications after surgery, polypropylene mesh ranked first, and a tendency to reduce stoma complications prevailed; however, the results were not statistically significant. Moreover, Byers et al. considered that it can also lead to a significant inflammatory response, resulting in serious complications such as adhesions, erosions, or wound infections [14]. The European Hernia Society guidelines on the prevention and management of parastomal hernia strongly recommend the use of non-absorbable synthetic mesh for the prevention of parastomal hernia in elective permanent terminal colostomies and do not recommend the use of absorbable or biological mesh. This finding is consistent with our results [1].

Recently, various types of composites meshes have been developed, usually made of polypropylene-based coated or double-layered materials, which combine the dual advantages of high strength and anti-adhesion properties. Polypropylene/PVDF, polypropylene/polyglecaprone (Monocryl), polypropylene/PG910, and polypropylene/cellulose (ORC) were used in this study. Polypropylene/PG910 ranked first in reducing the incidence of parastomal hernia according to the AUCRA; however, another composite mesh showed a decreasing trend, which was not statistically significant. Furthermore, based on the results of a meta-analysis by Network B, composite meshes of these absorbable materials did not significantly reduce other stoma-related complications. The findings of Emans et al. are consistent with our results, which show that the absorbable composite mesh has a good anti-adhesion effect in the short term; however, the anti-adhesion effect gradually decreases with the gradual absorption of its surface coating over time [32]. Titanium-coated polypropylene meshes are a novel type of composite mesh [33]. The non-absorbable feature of this mesh makes it possible to play a long-term role; yet the advantages and disadvantages of other aspects still lack sufficient research.

Collagen-based biological meshes were first introduced in the 1980s to identify the devastating consequences of mesh infection [34]. Biological meshes are prepared from human, bovine, or porcine decellularised collagen matrices, which are slowly degraded and replaced by fibrous tissue. Theoretically, once implanted, they become vascularised and cause migration of host cells; thus, they are less susceptible to infection [15]. In our study, the xenogeneic acellular dermis (porcine/bovine) biological mesh ranked second in reducing stoma-related complications according to SUCRA. In network A, the xenogeneic acellular dermis (porcine/bovine) biological mesh did not significantly reduce the incidence of parastomal hernia, which may be because the follow-up time of our included RCTs was > 12 months. Some studies have shown that the short-term curative effect of the mesh in the repair of incisional and parastomal hernias is good, and the long-term results of using porcine dermal collagen bio-mesh to repair PSH are unsatisfactory because of the high recurrence rate [35].

In a subgroup analysis comparing laparoscopic and open techniques, mesh placement may be more beneficial using the open technique, which is consistent with previous findings [36, 37]. However, it should be emphasised that the number of open cases in the included RCTs was much larger than that of laparoscopic cases, which may be the reason for the large heterogeneity. Depending on the location of the prophylactic mesh placement, routinely used methods include sublay, preperitoneal, and intraperitoneal methods. In the study of prophylactic mesh placement in terminal colostomy, no RCT trials were there on the placement of mesh between layers; however, most studies have focused on the use of sublay and intraperitoneal repairs. In subgroup analysis, the sublay repair method was statistically significant in preventing the occurrence of PSH. This result was consistent with the findings of Mohiuddin et al. [38].

However, this study has some limitations. Firstly, the number of RCT included in the network was small and the number of applications of several mesh materials was limited, which may have led to certain limitations in the network meta-analysis. Secondly, owing to the limitations of the intervention measures, some of the included studies could not be blinded and biases might have been there in implementation and measurement. Therefore, the results of this study should be interpreted with caution. Finally, further research on the controversial topic of which mesh material should be used as a prophylactic mesh requires additional RCTs to directly compare the effects of different mesh materials.

## Conclusion

Based on the network meta-analysis and ranking results, the polypropylene mesh material has better performance in comprehensively reducing the incidence of postoperative PSH and anastomotic complications, and it may be a better choice as a mesh material for preventive mesh placement. However, this conclusion needs to be confirmed with larger sample sizes and high-quality RCTs.

## Authors’contributions

CM, QW, and LS designed the research; CM and WQ collected the data; JG, YL, and XZ performed the data analysis; PW and LS wrote the manuscript; ZY revised the manuscript. ZZ and HY reviewed the manuscript. The authors have read and approved this manuscript.

### Supplementary Information

Below is the link to the electronic supplementary material.

Supplementary file1 (PDF 170 KB)

Supplementary file2 (PDF 170 KB)

Supplementary file3 (PDF 5 KB)

Supplementary file4 (PDF 5 KB)

Supplementary file5 (DOCX 11 KB)

Supplementary file6 (XLSX 11 KB)

Supplementary file7 (XLSX 10 KB)

## Data Availability

The datasets used and/or analyzed during the current study are available from the corresponding author on reasonable request.
