# Supplementary figures and images for: Effects of different mesh materials on complications after prophylactic placement for stoma formation: a systematic review and network meta-analysis
Source: Hernia. 2024 Jun 15;28(4):1039–52. doi: 10.1007/s10029-024-03068-y (PMC11297115; doi:10.1007/s10029-024-03068-y)

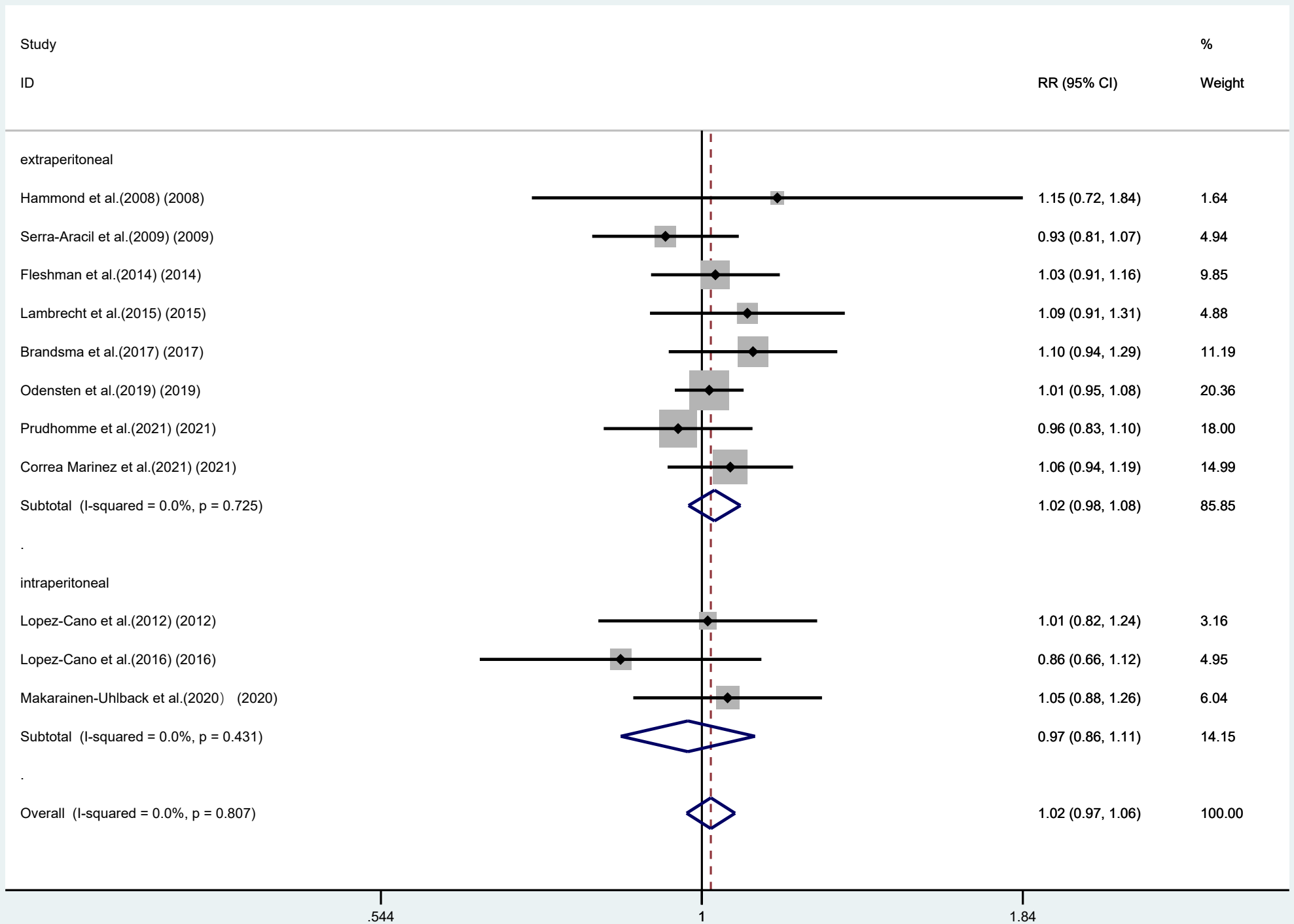

Supplement: Supplementary file 1 — Supplementary file1 (PDF 170 KB) [file 10029_2024_3068_MOESM1_ESM.pdf]

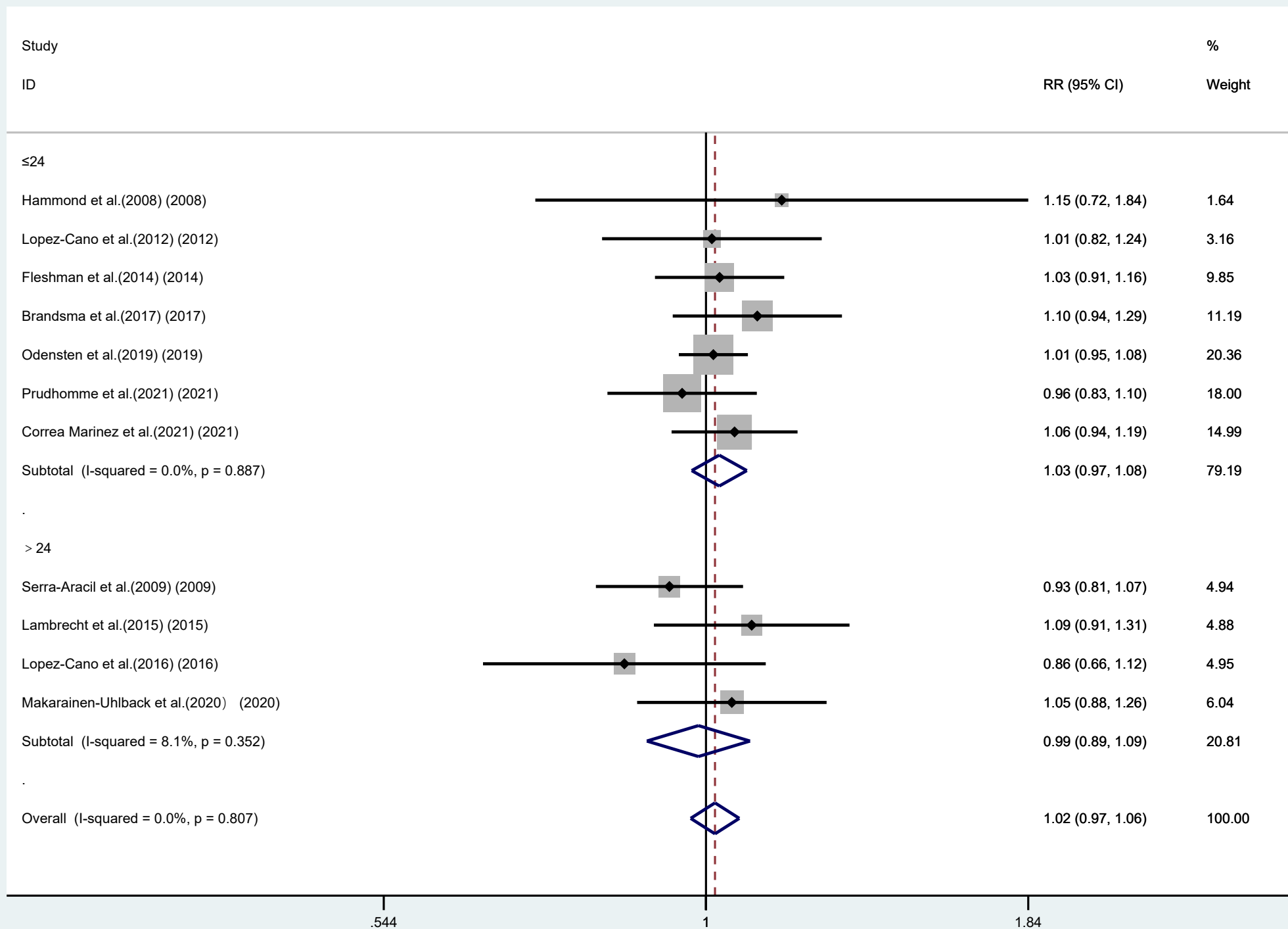

Supplement: Supplementary file 2 — Supplementary file2 (PDF 170 KB) [file 10029_2024_3068_MOESM2_ESM.pdf]

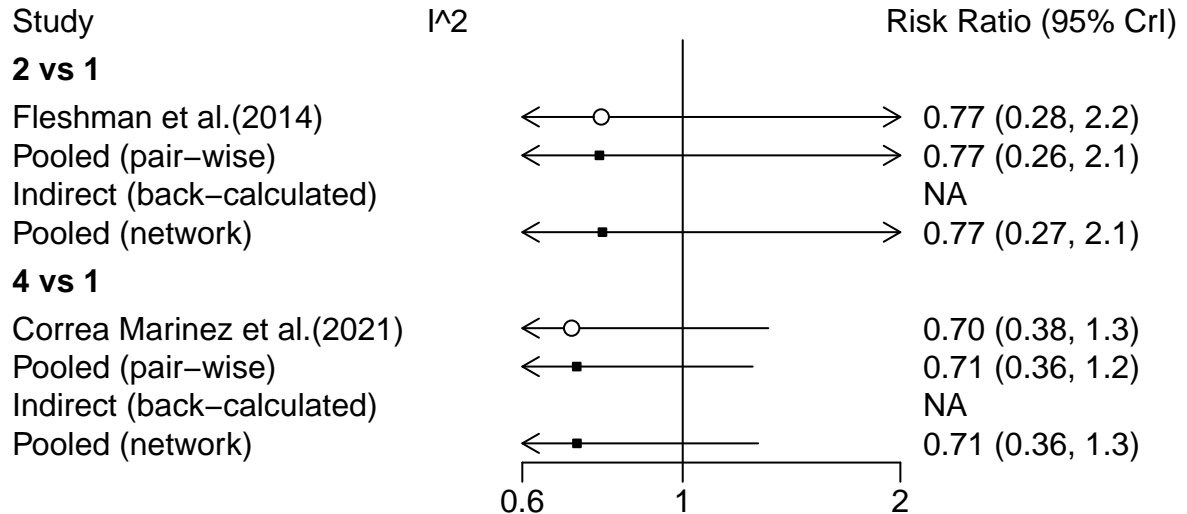

Supplement: Supplementary file 3 — Supplementary file3 (PDF 5 KB) [file 10029_2024_3068_MOESM3_ESM.pdf]
