# Supplementary material for: Effects of different mesh materials on complications after prophylactic placement for stoma formation: a systematic review and network meta-analysis
Source: Hernia. 2024 Jun 15;28(4):1039–52. doi: 10.1007/s10029-024-03068-y (PMC11297115; doi:10.1007/s10029-024-03068-y)
Supplement: Supplementary file 4 — Supplementary file4 (PDF 5 KB) [file 10029_2024_3068_MOESM4_ESM.pdf]

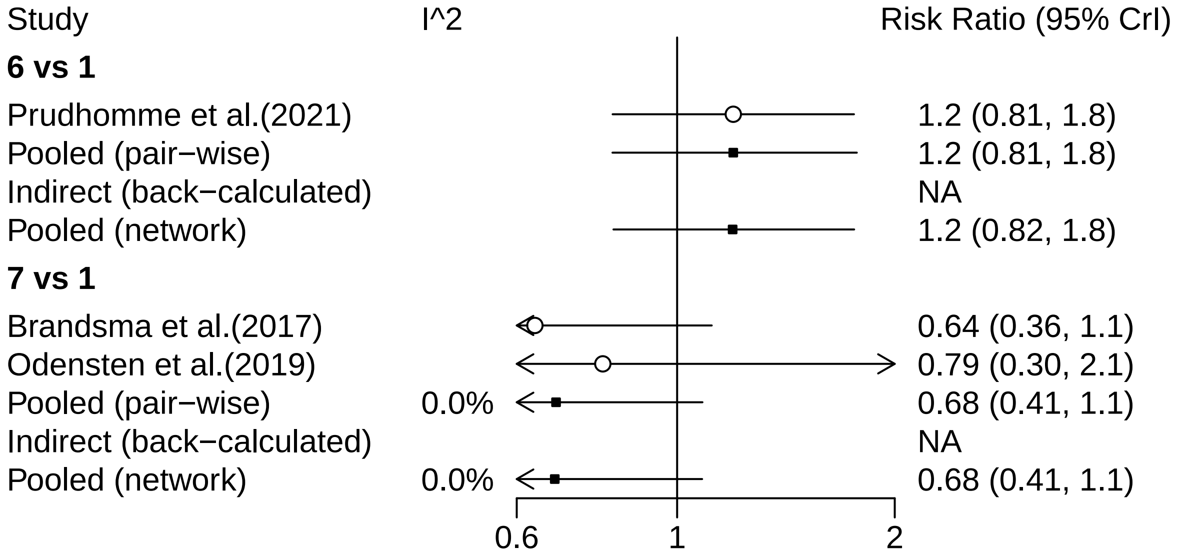
Study
I^2
Risk Ratio (95% CrI)
6 vs 1
Prudhomme et al.(2021)
1.2 (0.81, 1.8)
Pooled (pair−wise)
1.2 (0.81, 1.8)
Indirect (back−calculated)
NA
Pooled (network)
1.2 (0.82, 1.8)
7 vs 1
Brandsma et al.(2017)
0.64 (0.36, 1.1)
Odensten et al.(2019)
0.79 (0.30, 2.1)
Pooled (pair−wise)
0.0%
0.68 (0.41, 1.1)
Indirect (back−calculated)
NA
Pooled (network)
0.0%
0.68 (0.41, 1.1)
0.6
1
2
