# Supplementary material for: Effects of different mesh materials on complications after prophylactic placement for stoma formation: a systematic review and network meta-analysis
Source: Hernia. 2024 Jun 15;28(4):1039–52. doi: 10.1007/s10029-024-03068-y (PMC11297115; doi:10.1007/s10029-024-03068-y)
Supplement: Supplementary file 5 — Supplementary file5 (DOCX 11 KB) [file 10029_2024_3068_MOESM5_ESM.docx]

1. ****g. :****

****PUBMED****

**(((((((((colostom*[Title/Abstract]) OR (ileostom*[Title/Abstract])) OR (enterostom*[Title/Abstract])) OR (stom*[Title/Abstract])) OR (ostom*[Title/Abstract])) OR (parastom*[Title/Abstract])) OR (Enterostomy[MeSH Terms])) OR (Surgical Stomas[MeSH Terms])) AND ((hernia*[Title/Abstract]) OR (Hernia, Abdominal[MeSH Terms]))) AND ((((mesh*[Title/Abstract]) OR (prosthesis*[Title/Abstract])) OR (implant*[Title/Abstract])) OR ((((Surgical Mesh[MeSH Terms]) OR (Prosthesis Implantation[MeSH Terms])) OR (Absorbable Implants[MeSH Terms])) OR (Bioprosthesis[MeSH Terms])))**

****COCHRANE****

**(((parastom*) OR (PSH)) OR (parastomal hernia)) AND (((((prevention) OR (prophylactic mesh)) OR (prophylaxis)) OR (mesh)) OR (surgical mesh))**
